# Electronic Nicotine Delivery Systems and E-Liquid Modifications to Vape Cannabis Depicted in Online Videos

**DOI:** 10.1001/jamanetworkopen.2023.41075

**Published:** 2023-11-02

**Authors:** Rachel R. Ouellette, Sophia Selino, Grace Kong

**Affiliations:** 1 Department of Psychiatry, Yale School of Medicine, New Haven, Connecticut; 2 The Consultation Center, Yale School of Medicine, New Haven, Connecticut

## Abstract

**Importance:**

Electronic nicotine delivery systems (ENDS) were created to vape nicotine e-liquids; however, social media demonstrates increased ENDS modifications to vape cannabis. Analysis of social media content helps with understanding ENDS modifications for cannabis use, overlapping markets for ENDS and cannabis, and the need for additional regulation.

**Objective:**

To analyze online videos for use, modification, and marketing of ENDS to vape cannabis.

**Design, Setting, and Participants:**

In this qualitative study, 10 searches were performed on YouTube on July 14, 2022, using different combinations of relevant search terms to identify videos depicting modifications to ENDS and e-liquids to vape cannabis. The first 40 videos from each search (400 videos total, 152 without duplicates) were extracted, of which 59 included depiction or discussion of cannabis vaping content. Content analysis of videos included iterative development of codes, consensus building across team members, and reliability checks.

**Main Outcomes and Measures:**

Metadata (ie, video length, number of likes, comments, and views) were extracted for included videos. Coding was performed for uploader type, modifications to ENDS and e-liquids, devices used, cannabis products vaped, age restriction, purchasing links, health claims, and discussion of warning messages and cannabis regulations.

**Results:**

Of the 59 videos analyzed, 34 (58%) included tetrahydrocannabinol; 33 (56%), cannabidiol; and 8 (14%), both. A total of 21 videos (36%) were age-restricted, while 25 (42%) included purchasing links for cannabis products, 20 (34%) for ENDS, and 7 (12%) for nicotine e-liquids. Twenty-six videos (44%) made claims about health benefits of vaping cannabis. Forty-one videos (69%) demonstrated vaping manufacturer-made cannabis products (eg, cannabis concentrates) with no modifications needed. Eighteen videos (31%) depicted modifications to e-liquids or ENDS for cannabis use. The most frequent modification was mixing e-liquids with cannabis products to be compatible with ENDS. The most frequently reported reason for modifications was to vape cannabis in public by using the same devices used for nicotine.

**Conclusions and Relevance:**

This qualitative study of online videos found high frequencies of statements about health benefits from vaping cannabis, frequent purchasing links, and inadequate age restrictions. Exposure to such content on social media platforms may increase risk of cannabis vaping among youth. Use of the same devices for multiple substances may also enable dual use and complicate the US Food and Drug Administration’s regulation of ENDS.

## Key Points

**Question:**

What modifications to electronic nicotine delivery systems (ENDS) to vape cannabis are depicted on YouTube?

**Findings:**

This qualitative study of 59 online videos found that 41 videos (69%) depicted use of ENDS to vape cannabis without modifying devices or e-liquids by using manufacturer-made cannabidiol or tetrahydrocannabinol oils and/or e-liquids compatible with ENDS; the most frequent modification was creating e-liquids from cannabis concentrates and dry herbs. Only 21 videos (36%) were age-restricted, while 25 (42%) included purchasing links for cannabis products.

**Meaning:**

These findings suggest that vape shops and cannabis suppliers are creating products compatible with ENDS, enabling use of similar devices for nicotine and cannabis.

## Teaser

This qualitative study assesses the content of YouTube videos showing use and modifications of electronic nicotine delivery systems to vape cannabis.

## Introduction

Electronic nicotine delivery systems (ENDS) are devices created to turn e-liquids into aerosol using a heat source. They are the most frequently reported nicotine product used by middle and high school students.^1^ E-liquids consist of a mixture of propylene glycol and vegetable glycerin and typically contain nicotine and flavoring. However, ENDS are being increasingly used by adolescents to inhale cannabis,^2^ particularly cannabis concentrates including liquid distillates of tetrahydrocannabinol (THC) and cannabidiol (CBD) (ie, cannabis oils) and wax-based cannabis products like butane honey oil concentrate.^3^ Vaping cannabis is associated with increased risk of respiratory symptoms (eg, wheezing and bronchitis),^4^ due in part to the unregulated nature of cannabis concentrates, which can contain dangerous chemicals and additives contributing to ENDS-associated or vaping-associated lung injuries.^5^ Cannabis concentrates also contain high concentrations of synthetic cannabinoids,^6^ which can increase health risks (eg, cognitive impairments), particularly among adolescents.^7^

### ENDS at the Intersection of Cannabis and Nicotine Use Among Youth

ENDS use for nicotine is associated with increased risk of cannabis vaping, particularly among youth, including adolescents (ie, aged 10-18 years) and young adults (ie, aged 18-25 years).^8,9,10^ Among adolescents who vape cannabis, high proportions report using devices identified as ENDS to vape cannabis liquids.^11^ Given that ENDS were created to vape nicotine, adolescents may be using these devices for purposes and products that they were not intended for. However, it is unclear what modifications to ENDS and e-liquids are being made to vape cannabis.

The dual use of ENDS to vape nicotine and cannabis creates unique considerations for regulatory bodies such as the US Food and Drug Administration Center for Tobacco Products (FDA CTP). Although the FDA CTP does not regulate cannabis products, it regulates “the manufacture, import, packaging, labeling, advertising, promotion, sale, and distribution of tobacco products, including ENDS.”^12^ To inform the FDA CTP’s regulation of ENDS, it is important to understand how cannabis products are currently used in these devices. It is also unclear whether overlapping devices for nicotine and cannabis has resulted in intersecting markets where ENDS and cannabis products are sold together.

### Understanding ENDS Modifications Through Online Video Content

YouTube is a social media platform used to view, share, like, and comment on videos of varying length. The platform’s video format makes it highly suitable for tutorial-style videos depicting ENDS modifications, as seen in previous content analyses identifying frequent videos teaching viewers how to use ENDS, conduct vape tricks, and “hack” or modify devices.^13,14^ Massey et al^15^ conducted a content analysis of ENDS modifications on YouTube, where 3.6% of videos depicted adding other substances, including cannabis, to e-liquids. Lim et al^16^ found increasing frequencies of cannabis vaping content on the platform between 2016 and 2020, including high numbers of videos demonstrating how to use and maintain vaporizers and create cannabis e-liquids. However, their study did not examine specific ENDS and e-liquids modifications to vape cannabis. Thus, content analysis of YouTube videos presents a unique opportunity to understand what modifications, products, and product features enable use of ENDS to vape cannabis.

Online video content can also influence youth cannabis use by altering their knowledge and perceptions of ENDS for vaping cannabis. Previous examination of cannabis and ENDS content on YouTube highlights high frequencies of positive claims about vaping cannabis,^17^ which can influence adolescents’ decisions to use these products.^18^ YouTube policies currently impose age restrictions on “harmful or dangerous activities, including regulated substances and drugs,” including videos “promoting a cannabis dispensary” and “reviewing brands of nicotine e-liquid.”^19^ Despite these regulations, Lim et al^16^ found that 52% of identified cannabis vaping videos were not age-restricted. Additionally, more than 50% of cannabis-related videos on the platform include external purchasing links.^20^ This is particularly concerning because 95% of adolescents in 2022 reported using YouTube, making it the most frequently used social media platform among youths between 13 and 17 years of age.^21^

## Methods

In this qualitative study, we conducted a content analysis of YouTube videos showing use and modifications of ENDS to vape cannabis. We coded videos for types of modifications, reasons for modifying and using ENDS to vape cannabis, inclusion of THC and/or CBD, presence of age restriction and purchasing links, positive or negative health claims, inclusion of warning messages, and discussion of cannabis laws and regulations.

This study was exempt from institutional review board approval and informed consent in accordance with the Common Rule, as all data were publicly available. The study followed the Standards for Reporting Qualitative Research (SRQR) reporting guideline.

### Procedures

On July 14, 2022, we searched YouTube using search terms *how to* or *DIY* videos discussing modifications or *hacks* to *e-liquid* or *e-juice* to *vape cannabis*, *CBD*, or *THC*. We ran 10 searches total using different combinations of search terms (eg, *e-liquid hack THC*), restricted to videos in English. We extracted the first 40 videos from each search according to the platform’s default relevance algorithm^22^ (400 videos total, 152 without duplicates). We screened each unique video to confirm for cannabis vaping content, resulting in 59 eligible videos (Figure).

**Figure.**
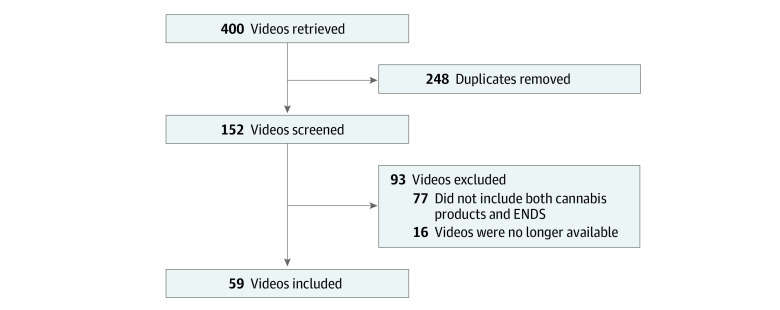
Flowchart of Included Videos ENDS indicates electronic nicotine delivery systems.

### Qualitative Approach

#### Content Analysis

We qualitatively examined and coded each video using content analysis through the development and application of a codebook containing both inductive and deductive codes. Inductive codes were developed by 2 team members (R.R.O. and S.S.) with feedback from the senior author (G.K.) through an iterative process of viewing 5 randomly selected videos, making note of common ENDS and e-liquids modifications to vape cannabis, reasons for modification, and statements about health risks or benefits and cannabis regulations. This process continued (randomly selecting additional sets of 5 videos) until saturation was met (ie, no new codes identified). We combined these inductive codes with deductive codes used previously by the study team to examine uploader characteristics and ENDS device types.^13,20^ Once the codebook was finalized, the first 2 authors coded 20 videos together to establish consensus. Coders were allowed to view each video and video description as many times as needed. After establishing reliability (>80% agreement), the first author (R.R.O.) coded all videos and the second author (S.S.) double-coded 30 randomly selected videos not used for consensus (82.3% agreement). Coding was initially conducted in Excel, version 2307 (Microsoft Corp), with final codes entered into SPSS, version 29 (IBM Corp). Code frequencies and descriptive statistics (median [IQR], minimum, and maximum) for metadata (ie, video date and duration and numbers of views, likes, and comments) were conducted in SPSS, version 29.

#### Content Areas

eTable 1 in Supplement 1 provides detailed descriptions of each content area coded across 59 videos. Content areas include metadata (eg, number of views, likes, and comments); uploader type (eg, vape shops, nicotine and/or cannabis enthusiasts, independent users of the platform); perceived demographic characteristics, including age, gender, and race or ethnicity (which were collected to evaluate whether specific viewers may be targeted in marketing posts via use of messengers with similar demographic characteristics); types of e-liquid and device modifications and reasons for modifying; devices used for cannabis vaping (eg, vape pens, box mods, pod systems); e-liquid contents (eg, nicotine, flavors, CBD, THC); age restriction, purchasing links, and discount codes; health risks or benefits endorsed by video uploaders; and inclusion or discussion of cannabis warning messages or laws and regulations.

## Results

A total of 59 videos were included in the analysis; 34 (58%) depicted tetrahydrocannabinol; 33 (56%), cannabidiol; and 8 (14%), both. Table 1 includes all metadata across videos. Table 2 includes frequencies for each content area across all videos and separately for videos with THC or CBD. eTable 2 in Supplement 1 provides links and descriptions of 5 coded videos. Herein, we describe the modifications made, reported health claims, and inclusion of warning messages for cannabis vaping.

**Table 1.** Descriptive Statistics of Online Videos Depicting ENDS Use and Modifications for Vaping Cannabis

Video characteristics	Median (IQR)	Minimum value	Maximum value
Published date	May 7, 2018 (November 8, 2016, to May 16, 2019)	June 27, 2014	April 6, 2022
Video duration	4 min 6 s (77-609 s)	14 s	75 min 10 s
No. of views	5720 (1025-213 734)	39	3 030 664
No. of likes	35 (6-1696)	0	37 130
No. of comments	12 (1-221)	0	4606

Abbreviation: ENDS, electronic nicotine delivery systems.

**Table 2.** Content Areas Coded Across Online Videos Depicting Cannabis Vaping^a^

Content	Type of video		
	All (n = 59)	THC (n = 34)	CBD (n = 33)
Uploader type^b^			
Cannabis enthusiasts	23 (39)	19 (56)	8 (24)
Private users	14 (24)	5 (15)	11 (33)
Online vape shops	11 (19)	4 (12)	9 (27)
Vape enthusiasts	4 (7)	2 (6)	2 (6)
Brick-and-mortar vape shops	3 (5)	1 (3)	2 (6)
New channels	3 (5)	2 (6)	1 (3)
Medical community	1 (2)	1 (3)	0
Other	1 (2)	0	1 (3)
Perceived gender of video participants^c^			
Female	9 (14)	5 (14)	4 (12)
Male	43 (68)	29 (78)	19 (56)
Cannot identify	11 (17)	3 (8)	11 (32)
Perceived race or ethnicity of video participants^c^			
Asian or Asian American	1 (2)	0	1 (3)
Black or African American	2 (3)	1 (3)	2 (6)
Hispanic or Latino	0	0	0
White or European	39 (62)	25 (68)	17 (50)
>1 race or ethnicity	5 (8)	4 (11)	1 (3)
Cannot identify	16 (25)	7 (19)	13 (38)
Perceived age of video participants, y^c^			
<18	0	0	0
18-24	7 (11)	4 (11)	3 (9)
25-34	13 (21)	8 (22)	5 (15)
35-59	14 (22)	9 (24)	8 (24)
≥60	1 (2)	0	1 (3)
Cannot identify	28 (44)	16 (43)	17 (50)
Modifications			
Any modification	18 (31)	17 (50)	3 (9)
Creating own e-liquids	13 (22)	12 (35)	2 (6)
Modifying devices or cartridges	5 (8)	5 (15)	1 (3)
Device type			
Vape pens	27 (46)	19 (56)	13 (39)
Cartridges	27 (46)	19 (56)	13 (39)
Box mods	14 (24)	7 (21)	8 (24)
Coil atomizers	9 (15)	5 (15)	4 (12)
Pod systems	7 (12)	3 (9)	3 (9)
Vaping kits	3 (5)	0	3 (9)
Cigalikes	0	0	0
E-liquid contents			
CBD	33 (56)	9 (27)	NA
THC	34 (58)	NA	9 (27)
Nicotine	1 (2)	0	1 (3)
Flavors	19 (32)	7 (21)	12 (36)
Terpenes	7 (12)	6 (18)	1 (3)
PG and/or VG	11 (19)	7 (21)	5 (15)
Age-restricted	21 (36)	20 (59)	5 (15)
Products advertised or sold via purchasing links or discounts			
Vaping devices	20 (34)	10 (29)	12 (36)
Cannabis products	25 (42)	6 (18)	22 (67)
Flavored e-liquids without cannabis	8 (14)	3 (9)	5 (15)
Nicotine products	7 (12)	3 (9)	5 (15)
Accessories for mixing e-liquids	6 (10)	6 (18)	2 (6)
Wax liquidizer	6 (10)	5 (15)	1 (3)
Merchandise	2 (3)	1 (3)	2 (6)
Health claims			
Reported health benefits	26 (44)	12 (35)	19 (58)
Reported health risks	11 (19)	7 (21)	5 (15)
Warning messages and regulations			
Cannabis warning message	6 (10)	4 (12)	3 (9)
Discussion of cannabis laws	14 (24)	10 (29)	6 (18)

Abbreviations: CBD, cannabidiol; NA, not available; PG, propylene glycol; THC, tetrahydrocannabinol; VG, vegetable glycerin.

^a^ Data are expressed as No. (%) of online videos.

^b^ Categories are not mutually exclusive. One CBD Uploader is both a brick-and-mortar vape shop as well as an online vape shop, making the total number 34.

^c^ Uploader-perceived demographic variables include 63 people in all videos (37 in THC videos and 34 in CBD videos) due to some videos containing multiple individuals. These variables were coded based on physical appearance and may not be accurate. Perceived demographic characteristics were collected to evaluate whether specific viewers may be targeted in marketing posts via use of messengers with similar demographic characteristics.

### Modifications

In total, 18 videos (31%) depicted modifications to ENDS or liquids to vape cannabis. Five videos depicted modifications to ENDS (eg, extracting liquid from broken cartridges, reusing disposable cartridges by refilling them with CBD or THC distillates, or placing cannabis wax directly on atomizer coils in devices meant for e-liquid), and 13 videos (22%) with modifications showed the video uploader making their own cannabis e-liquid. Five of these videos showed the uploader making liquid from THC or CBD oil or distillates; 5 showed making liquid from cannabis concentrates such as shatter, wax, or butane honey oil (ie, condensed products derived from cannabis plants to contain high concentrations of THC); and 3 showed the uploader making liquid from dry herb by heating it into rosin, a pressurized and condensed version of cannabis. No videos demonstrated creating e-liquids to contain both nicotine and cannabis. The most frequent reasons reported by video uploaders for mixing their own cannabis liquids were to (1) create liquids that are compatible with devices used for nicotine to vape cannabis in public; (2) save money; (3) control contents to minimize impurities; and (4) improve the taste.

The remaining 41 videos (69%) depicted vaping cannabis products that required no modifications for use in ENDS, including (1) refilling reusable cartridges, pods, vape pens, and box mods using CBD and THC liquids and/or oils; (2) using specific cartridges compatible with ENDS that contain cannabis liquids; and (3) purchasing manufacturer-made attachments to box mod devices to vape cannabis wax and dry herb. Of note, it was frequently difficult to decipher whether CBD and THC liquids vaped in these videos were created for use in ENDS or if they were oil-based products created for other purposes (eg, tinctures consumed orally). Additionally, only 1 of the 34 videos (3%) depicting THC products specified that it contained Δ-8-THC. No other videos specified the type of THC (ie, Δ-8 vs Δ-9).

### Purchasing Links and Age Restrictions

Only 21 videos (36%) included age restrictions. Twenty-five videos (42%) included purchasing links for cannabis products, 20 (34%) for ENDS, and 7 (12%) for nicotine products.

### Reported Health Claims

Twenty-six videos (44%) made claims about health benefits from vaping cannabis (12 of 34 total videos with THC [35%] and 19 of 33 total videos with CBD [58%]). Eleven of the full set of 59 videos (19%) made claims about negative health effects of vaping cannabis (7 of 34 total videos with THC [21%] and 5 of 33 total videos with CBD [15%]).

The most common positive health effects reported across videos include mental health benefits (16 [27%]) for managing anxiety, depression, and attention difficulties; pain management (10 [17%]); benefits for other physical ailments, such as nausea, epilepsy, cancer, chest and lung conditions, and inflammatory diseases (10 [17%]); benefits for sleep (5 [8%]); facilitating nicotine vaping cessation by presenting a “healthier replacement” for nicotine and easing withdrawal symptoms (4 [7%]); CBD as nonaddictive with minimal adverse effects and withdrawal symptoms (2 [3%]); and general comments referencing THC or CBD as “medicinal” and “healing” (9 [15%]). The most common negative health effects reported by video uploaders were dangers from inappropriate use or modifications to devices that can lead to combustion and injury (4 [7%]); high-potency THC distillates that can increase risk for overconsumption, withdrawal, paranoia, and negative cognitive effects (eg, difficulty concentrating) (2 [3%]); establishing tolerance quickly, requiring higher doses with increased risk for adverse effects (2 [3%]); increased drowsiness that impedes ability to drive safely (1 [2%]); burning sensation in the nose and throat (1 [2%]); adverse reactions from combining cannabis and nicotine (1 [2%]); unintended adverse effects including amplified pain (1 [2%]); and lack of clarity about potential health risks (1 [2%]).

### Warning Messages and Cannabis Regulations

Six videos (10%) showed or discussed cannabis warning messages, with most including messages on labels stating that the product has not been evaluated by the FDA and is “not intended to diagnose, treat or cure any disease.” While some video uploaders showed these warning messages to viewers, they were often paired with statements demonstrating mistrust of the FDA, potentially undermining the impact of these warning messages. Fourteen videos (24%) also included written or verbal discussions of cannabis laws and regulations, including statements about the legal age for consumption and clarifications that they live in a state where cannabis is legal (example statements are provided in eTable 3 in Supplement 1). Twelve videos (20%), all of which focused on vaping THC, also included statements about how vaping cannabis using ENDS enables the consumption of cannabis in public where consumption would otherwise be illegal.

## Discussion

Content analysis of 59 videos depicting use of ENDS to vape cannabis highlights a minimal need for modifications to e-liquids or devices to vape CBD and THC. Eighteen videos (31%) included modifications, with most demonstrating how to mix e-liquids to contain CBD or THC and how to refill disposable cartridges with cannabis liquids or oils. The remaining 41 videos (69%) depicted use of manufacturer-made and ENDS-compatible CBD or THC cartridges or refilling reusable ENDS (eg, vape pens) with CBD or THC liquids or oils.

It is important to highlight that many of the oils used to refill cartridges, vape pens, and box mods were not specifically created for vaping. This includes CBD and/or THC oils created to be used in other ways (eg, tinctures consumed orally). This presents concerns about vaping products not originally developed or tested for inhalation. This can contribute to unintended injuries and negative health effects. In fact, a previous study^23^ found that 82% of patients with ENDS-associated or vaping-associated lung injuries reported using THC vaping products, and 16% reported using CBD vaping products. In a sample of US adolescents,^24^ those who vaped cannabis had increased odds of respiratory symptoms, such as wheezing and dry cough. Many of these injuries and negative health effects are the result of additives, such as vitamin E acetate, that are harmful when inhaled. Previous studies examining CBD and THC products^25,26^ found that product contents frequently do not match product label claims, including differing CBD or THC concentrates and unlisted chemicals.

Lack of quality control of cannabis oils becomes especially problematic for CBD products, which are not age restricted in many states but may contain higher levels of THC than the 0.3% legal limit.^25^ Emerging research offers chemical evidence that CBD has the potential to break down under high temperatures into other compounds, such as THC, making it potentially psychoactive when consumed using ENDS.^27^ Adolescents may therefore have access to CBD oils that have unintended psychoactive or health effects when vaped.

Regulation of cannabis vaping is particularly complicated, as it resides at the intersection of multiple regulatory bodies. The FDA CTP regulates ENDS under the Family Smoking Prevention and Tobacco Control Act, after the 2016 Deeming Rule providing FDA regulatory authority over ENDS, including “e-liquids, vials that contain e-liquid, cartridges, flavors, certain batteries, and even software.”^28^ These restrictions do not include cannabis products, such as CBD and THC; however, this line becomes blurred when ENDS are used to consume cannabis.

Additionally, Δ-9-THC is regulated under the Controlled Substances Act^29^; however, CBD, including CBD byproducts such as Δ-8-THC, was ruled exempt from the Controlled Substances Act after the Agriculture Improvement Act of 2018.^30^ In January 2023, the FDA concluded that its existing regulatory frameworks do not properly address CBD, highlighting the need for a new regulatory pathway.^31^ Still, all products, including CBD and THC, are included under the Federal Food, Drug, and Cosmetic Act,^32^ which stipulates that any product claiming therapeutic benefits is considered a drug and requires FDA approval. Our findings highlight a plethora of health claims, including by vape shops and CBD and THC distributors, about health benefits from vaping cannabis that have yet to be corroborated by adequate empirical evidence to receive FDA approval. Social media platforms may therefore be providing viewers with misinformation about health effects of vaping cannabis, potentially contributing to risky health decisions. When misleading content related to ENDS is disseminated by tobacco suppliers, the FDA CTP can issue warning letters to companies.^14^ The FDA CTP, however, does not have the ability to regulate social media content disseminated by the general population. Instead, social media platforms have developed their own policies to monitor and regulate misinformation and marketing of tobacco and cannabis products. Despite these efforts, misinformation^33^ and marketing of cannabis vaping products^16^ persist on these sites.

The differences in regulatory practices between THC and CBD are also reflected in YouTube’s use of age restrictions. Videos with THC were approximately 4 times as likely as videos with CBD to be age-restricted, likely due to lack of US federal legislation restricting CBD. Some states have created their own policies restricting age of purchase for CBD, complicating the platform’s capacity to develop and enforce policies that align with varying restrictions across state borders. Purchasing links were provided in 25 videos (42%) for cannabis products, 20 videos (34%) for ENDS, and 7 videos (12%) for nicotine products. Meanwhile, YouTube age restrictions are for individuals 18 years and younger, meaning that individuals between the ages of 18 and 20 years can access all content and purchasing links despite not being of legal age to purchase these products.

The presence of purchasing links for both ENDS and nicotine products in videos for cannabis vaping also highlights overlapping markets between nicotine and cannabis that can complicate the regulation of ENDS. This is reinforced by vape shops that frequently sell both nicotine and CBD products,^34,35^ including CBD products that resemble tobacco products.^36^ Meanwhile, within the current study, the most frequently reported reason for vaping cannabis using ENDS was to use cannabis publicly, enabled by the inability of others to determine whether someone is vaping nicotine or cannabis. The overlap in devices therefore enables individuals to circumvent restrictions around consuming cannabis in public, further complicating efforts to regulate both ENDS and cannabis products. This can also promote dual use of nicotine and cannabis, which introduces unique health risks including increased risk of both nicotine and cannabis addiction.^37^

### Limitations

This study has some limitations. When coding for videos containing THC, we combined both Δ-9-THC and Δ-8-THC, due in part to lack of specification within videos. Future studies would benefit from examining these 2 variations separately due to their differing regulatory pathways. Additionally, the sample captured in this study may not reflect use behaviors of the broader population; therefore, further population-level studies are needed to better understand use and modification of ENDS to vape cannabis. It is also not yet understood what the effect of viewing these videos may be on adolescents and their decisions to vape cannabis using ENDS. Future studies would benefit from applying experimental designs to assess youth perceptions of social media content on ENDS use and modification to vape cannabis.

## Conclusions

In this qualitative study, the findings of our content analysis of online videos suggest that ENDS modifications are often not needed to vape cannabis due to the presence of manufacturer-made THC and CBD liquids compatible with ENDS. We observed high frequencies of positive health claims by video creators supporting the use of cannabis vaping for mental (eg, anxiety and depression) and physical (eg, pain) ailments. These statements, combined with the presence of purchasing links and inadequate age restrictions, bring salient risks for youth exposed to cannabis vaping content on social media, potentially resulting in increased dual use of ENDS for nicotine and cannabis. The use of similar devices to vape multiple substances complicates the regulation of both ENDS and cannabis products, requiring examination and clarification of how to best regulate use of ENDS to vape cannabis.

## Notes

Supplement 1. **eTable 1.** Description of Content Areas Coded Across 59 Videos
eReferences
**eTable 2.** Example YouTube Video Links and Descriptions **eTable 3.** Example Deidentified Statements From Included Videos on Cannabis Regulations

Supplement 2.
Data Sharing Statement
