# Supplementary material for: Electronic Nicotine Delivery Systems and E-Liquid Modifications to Vape Cannabis Depicted in Online Videos
Source: JAMA Netw Open. 2023 Nov 2;6(11):e2341075. doi: 10.1001/jamanetworkopen.2023.41075 (PMC10623192; doi:10.1001/jamanetworkopen.2023.41075)
Supplement: Supplement 1. — eTable 1. Description of Content Areas Coded Across 59 Videos eReferences eTable 2. Example YouTube Video Links and Descriptions eTable 3. Example Deidentified Statements From Included Videos on Cannabis Regulations [file jamanetwopen-e2341075-s001.pdf]

Supplementary Online Content

Ouellette RR, Selino S, Kong G. Electronic nicotine delivery systems and e-liquid modifications to vape cannabis depicted in online videos. *JAMA Netw Open*. 2023;6(11):e2341075. doi:10.1001/jamanetworkopen.2023.41075

eTable 1. Description of Content Areas Coded Across 59 Videos

eReferences

eTable 2. Example YouTube Video Links and Descriptions

eTable 3. Example Deidentified Statements From Included Videos on Cannabis Regulations

This supplementary material has been provided by the authors to give readers additional information about their work.

© 2023 Ouellette RR et al. *JAMA Network Open*.

eTable 1. Description of Content Areas Coded Across 59 Videos

Content Area	Codes
Metadata	– Upload date<br>– Video uploader channel ID<br>– Video length<br>– Number of views, likes, and comments
Uploader Type[a]	– Brick and mortar or online vape shops<br>– Vape enthusiasts *(i.e., independent users not associated with a vape company with >50% channel content vape-related)*[1,2]<br>– Cannabis enthusiasts *(i.e., > 50% channel content cannabis-related)*<br>– Medical professionals *(i.e., anyone with certified medical credentials)*<br>– News Channels<br>– Private users posting about assortment of topics
Perceived Gender of Individual(s) in Video[b]	– Female<br>– Male
Perceived Race of Individual(s) in Video[b]	– White/Caucasian/European<br>– Black/African American<br>– Asian/Asian American<br>– Hispanic/Latino<br>– Mixed Race<br>– Cannot Identify
Perceived Age of Individual(s) in Video[b]	– Younger than 18<br>– 18-24<br>– 25-34<br>– 35-59<br>– 60 or older<br>– Cannot Identify
Modifications to E-Liquids	– Adding specific substances (e.g., CBD, THC) to manufacturer made e-liquids (with or without nicotine)<br>– Creating their own cannabis e-liquids
Modifications to Devices	– Altering devices to be compatible with cannabis products *(e.g., rewicking devices)*
Reasons for Modification[c]	– Improve taste<br>– Save money<br>– Get a stronger or quicker high<br>– Less potent smell than combustible marijuana<br>– Able to vape cannabis in public<br>– Compatibility with nicotine-based devices<br>– Perceived health benefits<br>– Able to control e-liquid contents

© 2023 Ouellette RR et al. *JAMA Network Open*.

Device Type	– Cigalike<br>– Vape pen<br>– Box mod<br>– Pod system<br>– Vape kit<br>– Other
Device Components	– Cartridges<br>– Coil atomizers
Modified versus manufacturer-made E-Liquids	– Pre-mixed *(i.e., manufacturer-made cannabis e-liquid or oil)*<br>– Self-mixed *(i.e., cannabis e-liquid created/mixed by video uploader)*
E-Liquid/Oil Contents	– CBD<br>– THC<br>– Other cannabis products (e.g., wax concentrates and dry herb)<br>– Vegetable glycerin or propylene glycol<br>– Terpenes<br>– Nicotine<br>– Flavors
Age-restricted[d]	– Age-restricted<br>– Not age-restricted
Marketing and Promotion Types	– Purchasing links<br>– Discount codes
Products Advertised and/or Sold	– Vaping devices<br>– Wax liquidizers<br>– Nicotine products<br>– Cannabis products<br>– Merchandise<br>– Other accessories for mixing (e.g., beakers)
Location/Country Products Sold From	– Within the United States<br>– Outside of the United States
Uploader-Stated Health Benefits of Vaping Cannabis	– Helps manage anxiety, depression, and attention difficulties<br>– Pain management<br>– Improves physical ailments including nausea, epilepsy, cancer, chest and lung conditions, and inflammatory diseases<br>– Improves sleep<br>– Facilitates quitting nicotine by easing withdrawal<br>– Non-addictive<br>– General statements about cannabis as “medicinal” or “healing”
Uploader-Stated Health Risks of Vaping Cannabis	– Device malfunction and explosion from modifications<br>– Risks from consuming high concentrations of THC<br>– Easy to establish tolerance<br>– Drowsiness<br>– Burning sensation in nose and throat

© 2023 Ouellette RR et al. *JAMA Network Open*.

	–	Adverse reactions from combining nicotine and cannabis
	–	Amplified pain
	–	Lack of clarity about health risks
Warning Messages and Regulations	–	Presence of cannabis warning messages *(e.g., indicating product not certified by the FDA)*
	–	Information provided about cannabis laws and regulations *(e.g., age restrictions, whether marijuana is legal in state video uploader lives in).*

Abbreviations: CBD, cannabidiol; FDA, US Food and Drug Administration; THC, tetrahydrocannabinol.

[a]Coded based on content from uploader channel page.

[b]Perceived demographic variables were coded based on physical appearance and may not be accurate. Perceived demographic characteristics were collected to evaluate whether specific viewers may be targeted in marketing posts via use of messengers with similar demographic characteristics.

[c]As communicated by video uploader.

[d]Indicates not accessible to viewers younger than 18 years; requires sign-in to view.

eReferences

1. Kong G, Laestadius L, Vassey J, et al. Tobacco promotion restriction policies on social media. *Tob Control*. Published online November 3, 2022. doi:10.1136/tc-2022-057348
2. Vogels EA, Gelles-Watnick R, Massarat N. Teens, social media and technology 2022. August 10, 2022. Accessed January 15, 2023. https://www.pewresearch.org/internet/2022/08/10/teens-social-media-and-technology-2022/

© 2023 Ouellette RR et al. *JAMA Network Open*.

eTable 2. Example YouTube Video Links and Descriptions

YouTube Video Link	Description of Video Content	Age-Restricted
https://www.youtube.com/watch?v=Yh1y_n5GQv0	Video marketing CBD and Delta-8 "vape additive" combined with purchasing link and statements about potential health benefits (e.g., "potent relief") and product features (e.g., "smooth flavor")	Not age-restricted
https://www.youtube.com/watch?v=EW0GraIg6qU	Video marketing CBD and THC vape cartridges combined with purchasing link and statements about potential health benefits (e.g., "Good For Treating: Depression, Autism, PTSD, & More!")	Not age-restricted
https://www.youtube.com/watch?v=uHsnETI525Q	Video demonstrating how to make cannabis e-liquids from THC powder and wax liquidizer	Age-restricted
https://www.youtube.com/watch?v=QodcR6XOquo	Video demonstrating how to make THC e-liquids from cannabis concentrates and marketing e-liquids containing terpenes	Not age-restricted
https://www.youtube.com/watch?v=4AvISpz3z_E	Video discussing ENDS battery types compatible with 510 cartridges for cannabis oil	Age-restricted

© 2023 Ouellette RR et al. *JAMA Network Open*.

eTable 3. Example Deidentified Statements From Included Videos on Cannabis Regulations

Written Versus Spoken Content	Example Statement
Written	"This video was shot in a location where Cannabis is legal. The materials used are not intended or sold for the purpose of making or modifying any illegal substance. The techniques used in this video can be used to make E juice from a wide range of concentrates."
Spoken	"This video is for information purposes only. I do not condone that anyone break the law, so please check with your local state and federal laws before making your own cannabis e-juice."
Written	"Recreational and medical marijuana use, cultivation, and manufacture are legal under the laws of [STATE VIDEO UPLOADER IS FROM]; however, the Content of the Videos and the Channel itself is for ENTERTAINMENT AND EDUCATIONAL PURPOSES ONLY. THE POSSESSION, USE, CULTIVATION AND DISTRIBUTION OF ANY AMOUNT OF MARIJUANA, INCLUDING SEEDS, IS ILLEGAL UNDER U.S. FEDERAL LAW, AS WELL AS MANY STATE LAWS. [VIDEO UPLOADER NAME] DOES NOT ADVOCATE OR ENDORSE THE CONTRAVENTION OF THE LAW."
Written	"[VIDEO UPLOADER NAME] are a product review and reference organization of the Recreational Cannabis industry. Since the legalization of cannabis or marijuana, there's been an explosion in the Recreation Market in both smokable cannabis products along with Cannabis related smoking and Vaping equipment. Here we will be exploring all of the commercially available products with short openings and reviews of concentrates cannabis and other marijuana or cannabis-infused items."
Written	"For adults of legal smoking age." – *regarding Delta-8 product with CBD*

© 2023 Ouellette RR et al. *JAMA Network Open*.
