# Supplementary material for: Electronic Nicotine Delivery Systems and E-Liquid Modifications to Vape Cannabis Depicted in Online Videos
Source: JAMA Netw Open. 2023 Nov 2;6(11):e2341075. doi: 10.1001/jamanetworkopen.2023.41075 (PMC10623192; doi:10.1001/jamanetworkopen.2023.41075)
Supplement: Supplement 2. — Data Sharing Statement [file jamanetwopen-e2341075-s002.pdf]

Data Sharing Statement

Ouellette. Electronic Nicotine Delivery Systems and E-Liquid Modifications to Vape Cannabis Depicted in Online Videos. *JAMA Netw Open*. Published November 02, 2023. doi:10.1001/jamanetworkopen.2023.41075

Data
Data available: Yes
Data types: Data (not involving human participants), Data dictionary
How to access data: Available upon request from corresponding author at rachel.ouellette@yale.edu.
When available: With publication

Supporting Documents
Document types: None

Additional Information
Who can access the data: Data will be made available to any who requests, however YouTube user and channel IDs will be removed to preserve anonymity.
Types of analyses: Any purpose.
Mechanisms of data availability: With investigator support.
Any additional restrictions: The author team will not share YouTube user or channel IDs from the identified videos to respect video uploader privacy, despite all data being publicly available on YouTube.
